# *Varroa destructor* Mites Can Nimbly Climb from Flowers onto Foraging Honey Bees

**DOI:** 10.1371/journal.pone.0167798

**Published:** 2016-12-12

**Authors:** David T. Peck, Michael L. Smith, Thomas D. Seeley

**Affiliations:** Department of Neurobiology and Behavior, Cornell University, Ithaca New York, United States of America; University of California San Diego, UNITED STATES

## Abstract

*Varroa destructor*, the introduced parasite of European honey bees associated with massive colony deaths, spreads readily through populations of honey bee colonies, both managed colonies living crowded together in apiaries and wild colonies living widely dispersed in natural settings. Mites are hypothesized to spread between most managed colonies via phoretically riding forager bees when they engage in robbing colonies or they drift between hives. However, widely spaced wild colonies show *Varroa* infestation despite limited opportunities for robbing and little or no drifting of bees between colonies. Both wild and managed colonies may also exchange mites via another mechanism that has received remarkably little attention or study: floral transmission. The present study tested the ability of mites to infest foragers at feeders or flowers. We show that *Varroa destructor* mites are highly capable of phoretically infesting foraging honey bees, detail the mechanisms and maneuvers by which they do so, and describe mite behaviors post-infestation.

## Introduction

The parasitic mesostigmatid mite *Varroa destructor* [1] is a highly damaging pest of both managed and wild colonies of European honey bees (*Apis mellifera*). The parasitism of the mites, and the spread of the viruses that they vector during their feeding [2,3], causes devastation in honey bee colonies. The exponential reproduction of the mites builds their population in a bee colony to extraordinary heights, causing the demise of most untreated host colonies within a few years [4,5]. *Varroa* infestation has been identified as the primary factor contributing to high overwinter colony mortality in some analyses [6,7]. Mites can spread through the bee population both vertically and horizontally. Vertical transmission occurs when honey bee colonies cast reproductive swarms, and the phoretic mites travel upon the swarming bees to the new nest site. Horizontal transmission of mites between colonies is thought to take place primarily through drift of worker bees into colonies other than their own, robbing of honey stores from weak colonies by stronger ones, and the movement of infested brood or bees by beekeepers. However, these avenues of horizontal transmission may be absent or reduced in isolated wild colonies, such as the feral honey bee population of the Arnot Forest in New York State [8]. We investigated the plausibility of an additional horizontal transmission mechanism: mites passing between colonies by infesting foragers from a mite-free colony after having been groomed from infested bees and onto flowers.

There is already evidence that *Varroa* mites occasionally wind up on flowers. In 2000, a USDA inspector found a live mite in a refrigerated shipment of flowers from the Netherlands [9]. An exhaustive search of the flower shipment did not reveal any honey bees, suggesting that the mite had survived for an extended time on a flower. The authors speculated that such an infested flower placed in an open-air flower market might allow the spread of this mite across international borders. One author conducted “limited floral surveys” around honey bee colonies for *Varroa destructor*, but found none [9]. Another report recounts the discovery of a live mite on a dead honey bee contained in a shipment of cut flowers from South America [10]. The repeated detection of these international mite biocontainment breaches prompted our investigation into whether mites on flowers are capable of infesting a honey bee during natural foraging.

Research on the relationship between *Varroa destructor* and flowers has been modest. Hartwig and Jedruszuk [11] as well as Smirnov [12] showed that mites can survive on flowers for as long as six days, depending on flower species. Gromyko [13] maintained live *Varroa* on flowers for as long as six days under controlled conditions, and reported that some mites were able to climb onto dead bees presented to them afterwards on a watch glass. Hartwig and Jedruszuk [11] reported that some mites climbed onto live workers held against infested flowers for “about 30 seconds,” but the presentation of the bee to the floral mite was not naturalistic. Also, no investigation was made of anti-mite grooming by the foragers, and no information was reported about the mites' behavior during and after infestation. Thus, there exists suggestive evidence that *Varroa destructor* can move from flowers onto foraging bees, but there are few observations and no quantification of this phenomenon in a naturalistic context. As the mites lack eyes and likely rely on their chemosensory forelegs to detect potential hosts [14,15,16] we began this study with a genuine doubt that a mite on a flower would be capable of the sensory discrimination and rapid acrobatics required to detect and mount a foraging honey bee before it flew away.

Eickwort [17] described phoresy (one organism moving by attaching itself to another) as “the principal adaptation required of a mite in order to become an important associate of bees.” Schwarz & Huck [18] clearly demonstrated that floral waystations are used during bee-to-bee phoretic jumps by parasitic mites of bumble bees, and the phoretic interaction between mites, flowers, and hummingbirds has been elegantly described by Colwell [19]. It is unknown whether *Varroa jacobsoni*, the mites from which *Varroa destructor* recently evolved, displays behaviors that would enable mite transfer on flowers, but it is likely that these parasites have evolved a diverse range of phoretic dispersal mechanisms to move between widely spaced colonies of their host bee *Apis cerana*. The question which drove the present study was whether *Varroa destructor* mites can infest Western honey bees at flowers and so be carried to a new host colony, and whether this is a mechanism for mite dispersal between widely spaced colonies. We investigated mite behavior during flower-to-bee infestations, as mite success in these behaviors is critical to the floral transmission hypothesis. This study also helps us understand the nature of mite infestation of honey bees generally, as our methods allowed us to observe infestations in exquisite detail. We found that the behavior of mites on flowers results in efficient infestation of foragers, and that the behavior of mites on foragers results in avoidance of the honey bee’s behavioral resistance to mite infestation(i.e., grooming.)

## Methods

**Fig 1:**
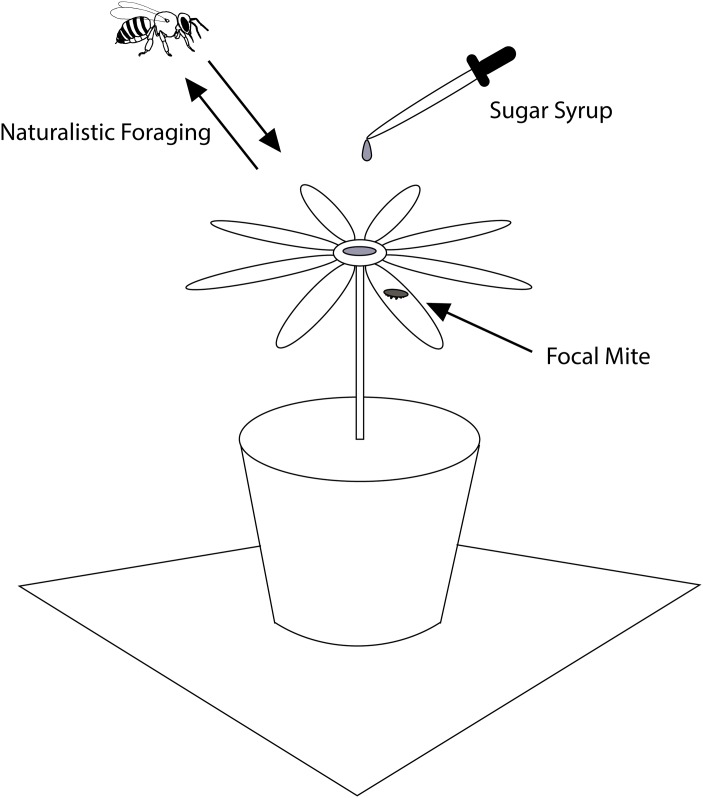
Experimental setup to monitor mite behavior towards foraging honey bees. To see whether mites fell off bees after climbing onto them, the top of the cup holding the focal flower was covered in white paper, as was the stool on which the cup sat. The entire apparatus was underlain by a 1m by 2m sheet of white cloth.

We conducted our studies at the Cranberry Lake Biological Station (44°09'N 74°48'W) in July of 2014. The station is owned by the State University of New York College of Environmental Science and Forestry, who generously allowed our work to proceed alongside their summer field training program. This field station is nestled in an extraordinarily nectar-poor area of the Adirondack State Park in northern New York State, where forager bees are easily trained to visit experimental food sources. To determine whether mites can infest foraging bees, we placed mites on focal flowers (Fig 1) or a sugar-water feeder (see Fig 4.5 in [20]) and observed the mites’ interactions with workers from a nearby honey bee colony that were visiting these food sources. The bees' arrivals and departures were recorded as were all mite orientations, movements, and interactions with the bees. We used both video recordings and direct observations to determine the behavior of both focal mites and honey bee foragers during each trial. Mites were placed on the grooved base of the feeder, on the yellow inflorescence or white petal of a daisy flower (*Bellis* cultivar), on the spikes in the center of an Echinacea flower (*Echinacea* cultivar), or on the petal of a speedwell flower (*Veronica* cultivar). White sheets of paper (22cmx28cm) and a white cloth (1mx1.9m) were spread underneath our equipment so we could detect any mites that fell off of bees within a half-meter radius of the focal flower or feeder.

Because our flowers provided an artificially rich food source in an otherwise forage-poor environment, each flower received much more forager attention than would be expected in nature. We cannot, therefore, consider the time that each mite spent on a flower before infesting a bee as a realistic indication of how long a mite waits to infest a forager in nature. Therefore, we report instead the total number of "bee-seconds" of forager activity experienced by a flower bearing a mite before the mite climbed onto a bee. This indicates the average amount of time a forager would spend on a mite-bearing flower before she becomes infested.

To maintain forager interest in the focal feeders and flowers, bees were offered sucrose solutions scented with anise extract. The molarity of the sugar solution was adjusted (0.25M to 2M) so that only one to four foragers visited the focal feeder or flower at any one time. Sucrose droplets were pipetted onto the center of all focal flowers, roughly at the position of the flowers’ own natural nectaries.

All bees were from a colony of European honey bees with a naturally mated queen. The colony occupied a ten-frame Langstroth hive body, with six frames that contained brood and were covered in adult bees, and four frames that held empty comb to motivate foraging. We selected this colony due to its easily portable size and its ability to provide us with plentiful mites to use in our tests(average mite density: 9.3 phoretic mites per 100 bees) as measured by sugar shake [21].

*Varroa* were obtained from the honey bee colony that also provided our foragers. We used the sugar shake method [21] to obtain live phoretic-stage mites. After removal from their bee hosts, each mite was removed from the powdered sugar with a toothpick, cleaned with chlorine-free water, examined for damage, and then placed in a shaded plastic container until being used in the experiment. Humidity in the container was maintained by a damp paper towel. No mite spent more than two hours in the container before being used in a trial.

## Results

### Infestation Success Rates

**Fig 2:**
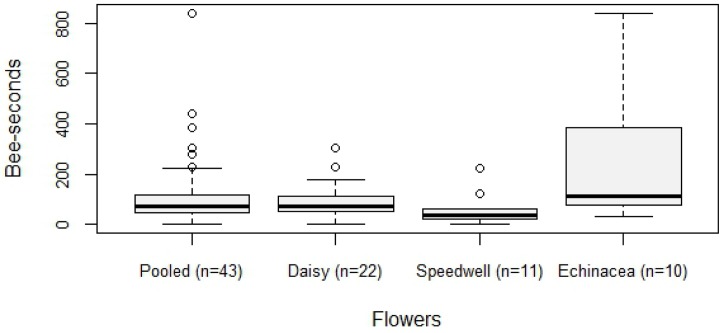
The number of bee-seconds before a honey bee forager was infested by the focal mite. Boxplots shown for each of the three species used, and pooled data from all three species. Boxes span the first through third quartiles, and whiskers span the range of non-outlier values.

Of 31 mites placed on the glass feeder, 29 infested a bee; the two that did not were blown off the feeder by wind. In 12 of the 29 infestations (40%), the bee immediately groomed herself, but in only 3 instances was the mite successfully dislodged. Consequently, 26 of the 31 mites (84%) left the feeder attached to a bee. Of 43 mites placed on flowers, all 43 infested a bee, and almost every one (41 of 43) left the flower on the bee it had infested. One mite fell off its forager, and one was groomed off. The average time taken by a mite placed on a flower to infest a foraging bee was 119 bee-seconds (Fig 2). The most rapid infestation from a flower took only 2 bee-seconds, while the longest took 840 bee-seconds.

When parsing the pooled flower data by flower species, we found that mites on Echinacea flowers experienced significantly more bee-seconds of foraging before infestation (243.1 +/- 253.9) compared to mites on daisies (92.6 +/- 72.6) (t_30_ = 2.60; P = 0.014) but not compared to mites on speedwell (59.1 +/- 63.8) (t_19_ = 2.33; P = 0.031) using a Bonferroni-corrected alpha of 0.016. Mites on daisies and speedwell were not significantly different from one another (t_31_ = 1.30; P = 0.204). Mites tested on daisy flowers were placed alternately on either the yellow center of the flower head or the white “petals” (ray florets). The bee-seconds before infestation did not differ significantly between these two groups (103.5 +/- 25.4 vs. 83.5 +/- 19.5) (t_20_ = 0.12; P = 0.908). The behaviors of the mites during infestations on each flower type were not discernably different and so were pooled with the feeder observations and videos for further analysis.

Approximately half of the mites, both on feeder (59%) and on flowers (49%), required only one contact with a bee before infesting it. The mean number of mite-bee contacts prior to and including infestation on the feeder and flowers was 2.1. The most mite-bee contacts any mite experienced was 7, most of which were contacts with the bee’s tarsal claw. Of the 22 out of 43 focal mites on flowers which did not infest a bee on first contact, 15 (68%) made contact with the tarsal claw of the bee on the first contact, and then infested the bee upon the next contact with a different body part.

Mites on both substrates (feeder or flowers) rarely walked more than one centimeter. Their activity level ranged from orientation and walking <2mm towards nearby foragers to standing still with no movement other than the occasional extension of the forelegs. The mites frequently engaged in the same repeated foreleg extension behavior: the chemosensory forelegs were extended forward and upwards. This behavior was infrequent when there were no bees on the forage, and increased in frequency when foragers moved to within a few centimeters of the mite. Some, but not all, mites oriented towards nearby foragers, and walked towards bees prior to infestation. Mites on flowers were frequently observed to move to the edges of petals and other floral structures and then remain facing outward. For example, mites placed on the center of Echinacea flowers universally moved to the tips of the spine-like disc florets and oriented their forelegs upwards.

### Mite Behavior During and After Infestation

**Fig 3:**
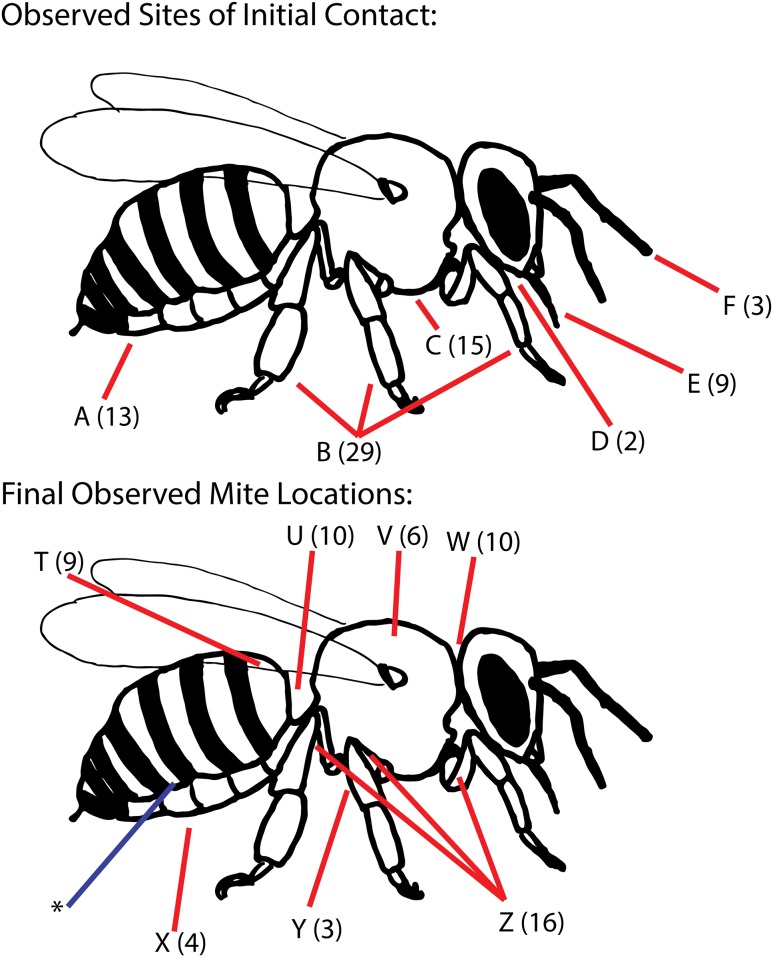
Sites of initial contact between foragers and mites (n = 71), and the sites at which mites came to rest (n = 58). Numbers in parentheses denote the number of mites observed interacting with each host body part. Data are pooled from feeder and flower observations. A: Any part of the ventral abdomen; B: Legs, above the tarsal claw; C: Ventral or lateral thorax or proximal surface of legs; D: Ventral surface of head; E: Proboscis; F: Antenna. T: Anterior dorsolateral abdomen, 1^st^ segment; U: Intertagmal region between thorax and abdomen (“waist”); V: Central dorsal thorax; W: Intertagmal region between head and thorax (“neck”); X: Ventral abdomen; Y: Ventral thorax; Z: Dorsal trochanter or femur. The asterisk refers to the space between the 3^rd^ and 4^th^ tergites: the most common location for *Varroa* found on hive bees in past studies.

Combining data obtained from observations on the feeder and the flowers, we saw 74 infestations. In 71, we observed the initial point of contact between bee and mite, and in 58 we observed the mite long enough to see the location on the bee upon which the mite stopped moving before the forager departed. The mounting of the foraging bees by the mites was rapid, and was followed immediately by movement from the site of first contact to one of a number of apparent refugia on the bee’s body (Fig 3). Analysis of 12 particularly detailed infestation videos showed that the mean time between the beginning of mite-bee contact and the mite coming to rest on the bee was extremely short, just 3.48 ± 1.85 s (range 1.58–6.98 s). Most initial contacts with the bee’s thorax and abdomen were with the ventral surface, meaning perhaps some of these infestations first observed on the thorax and abdomen may in fact have begun on the forager’s legs, which were the most common sites of first mite-bee contact. In every infestation, the first parts of the mite’s body to make contact with the bee were the extended forelegs, and in many cases mites were observed to latch onto a host with their forelegs and then rapidly (in < 0. 03 s) flip their body upside down to bring the rest of their legs in contact with the host. The locations where the mites settled (n = 58) most often were the dorsal intertagmal regions of the “neck” and “waist” (17% and 17%), the anterior dorsolateral portion of the first visible segment of the abdomen (16%), and the dorsal surfaces of the trochanter or femur (28%). Less common settling locations were the central dorsal thorax (10%), and the ventral surfaces of the thorax or abdomen (5% and 7%).

We did not observe the bees perform conspicuous avoidance behavior toward the mites at the feeder or the flowers. The bees did, however, respond to infestation; in many cases, foragers took off from the flower within one or two seconds of being infested. Of the 64 infestations in which we could evaluate grooming responses in real time or in the video recording, 20 bees (31%) began grooming themselves at the feeder or flower, though only 4 of these grooming events led to the mites detaching from the bee, and in 3 of those 4 cases the mite immediately mounted the same or a nearby forager. One instance of allogrooming was observed (on a flower), but the grooming bee failed to dislodge the mite from the groomed bee.

## Discussion

*Varroa destructor* mites are able to rapidly infest honey bees foraging at a feeder or at flowers of several species. Our observations reveal that mites can quickly mount honey bees engaged in foraging, and that despite efforts by the bees to groom off the mites, they almost always succeed in leaving the forage site still attached to a bee. *Varroa* transfer from flower to bee can occur in just 2 seconds of foraging activity on a flower. It is not yet clear how significant this mode of transmission may be for mite spread between colonies because little is known about how frequently mites wind up on flowers. Our study examined only the transfer of mites from flowers to bees but not from bees to flowers. Therefore our data are most relevant to situations such as those reports in the literature where live mites have been transported on flowers through biocontainment barriers [10;9], but they also support the plausibility of mite transfers between bee colonies via flowers. At this point, however, we do not claim that this is a common phenomenon.

We have also found that the behavior of *Varroa* upon infestation enables them to evade the bees’ grooming defense, giving them a chance to travel home with the hapless bee to her colony's nest, where they may be able to begin reproducing. Thorp [22] discusses similar “safe spaces” on various bee species which allow passively acquired pollen grains to evade bee grooming, and refers to Kimsey’s [23] work on mite exploitation of these same refugia from grooming in euglossine bees. *Varroa* success during the forager's flight back to the nest is unknown, though we monitored mite falls in a 1m radius around the forage source and across all of our observations fewer than 14% of our focal mites were recovered within this area. Mite success upon return to the hive is also unknown. Bees are also known to engage in allogrooming solicitation dances [24], which may or may not significantly reduce the survival of mites brought home by foragers.

Our use of mites from a single bee colony does not invalidate the conclusion that *Varroa* mites have the sensory and behavioral capacity to infest freely behaving forager bees at flowers. The colony from which we obtained our mites was selected not for any remarkable feature of its mites, but simply because it was a mite-laden colony in our bee yard which was small enough to be transported via boat to the study site. However, the fact that our focal mites and foraging bees were both from the same colony does mean that this study neglects possible effects of chemical adaptation between the mites and bees. *Varroa* mites have been shown to passively absorb the cuticular hydrocarbon profile of their host hive [25,26]. Therefore, a forager might have had an easier time detecting and removing a mite if the mite had the hydrocarbon profile of a foreign colony, (though mite grooming-avoidance behavior suggests increased detectability may not be sufficient to stop infestation.) Also notably, the mites in our study infested bees from their original hive instead of ignoring familiar-smelling hosts for the chance to disperse on a foreign bee. Further studies will be needed to tease apart whether or not colony-specific chemical cues play any part in *Varroa* infestation behaviors on flowers.

The behavior of the hostless mites that we observed suggests that they employ a “sit and wait” strategy, wherein little movement takes place except when cues from a nearby honey bee are detected. The foreleg extension behavior of the mites is comparable to the “questing” behavior seen in host-seeking ticks [27], but is difficult to assign definite function to it as the extension of the forelegs may serve a sensory or a mechanical purpose, or both. Mites perhaps use the chemosensory setae on their forelegs to “sniff” for potential hosts [14,15,16] or this foreleg extension may simply enable the mites to easily grasp a passing bee, or both. Once a bee approached, mites commonly but not universally oriented towards and approached the bee. More than half (53%) of the successful mites infested a forager upon first contact, while the other 47% made repeated contacts before successfully infesting a bee (usually the same one.) Most of the initial, unsuccessful contacts were between the mite and a tarsal claw of the bee, suggesting that tarsal claws lack the cues necessary to trigger mite behavior, that mites detect but do not attempt to climb the tarsal claws of honey bees, or that mites are unable to grab onto a bee’s tarsal claw when they are stepped on.

The mite behaviors that we observed during infestation show how foragers can become infested at flowers, but may also relate to infestations which take place in the nest. The initial refugia on forager bees that we observed (Fig 3) are not the sites that other investigators have reported for mites found on bees collected from colonies, but they may be the sites that provide combined safety from grooming and rapid accessibility. Perhaps we have identified the mites' preliminary refugia. The preferred feeding attachment site of *Varroa destructor* on bees in hives is between the third and fourth ventro-lateral tergites [28,29]. Whether mites move to our "preliminary refugia" during in-hive transfers between bees is unknown. It is currently unknown if the behaviors we observed are specially adapted for infesting bees engaged in foraging or are simply the typical first sites where a mite settles on any newly infested bee before moving to a preferred long-term location. Since mites prefer to use nurse bees as hosts over foragers [30,31,32], perhaps movement to these non-feeding refugia provides a safe place for a mite to wait until the forager it has infested brushes past a nurse bee in the hive. Kather and colleagues have shown that mites entering a new colony require a period of at least three hours to chemically adapt to the odors of their new hosts, [26] which offers another explanation for why mites on flowers would initially move to non-feeding sites which offer refuge from host grooming.

The actual likelihood of a mite transferring between two colonies via this floral transfer pathway is unknown, and further study is required to determine whether this is a major, minor, or completely negligible transmission phenomenon in nature. Optimal virulence theory predicts that parasites and pathogens should evolve lower virulence in systems wherein horizontal transmission is low relative to vertical transmission [33,34]. In a eusocial insect like the honey bee, the colony can be thought of as a superorganism, so colony-to-colony mite transmission can be considered horizontal transmission, and infestation persistence in mother swarms and daughter colonies can be considered vertical transmission. A complete understanding of both vertical transfer mechanisms (i.e. swarming) and horizontal mechanisms (e.g. drift of infested bees, robbing between colonies, floral transfers between foragers, etc.) is required before accurate predictions can be made about the direction of virulence evolution in the mites and mite-vectored viruses of honey bees living under natural conditions, (but see [35] for an excellent review of what is known). Floral transfer may represent an important avenue for horizontal mite transmission between widely spaced wild colonies. Whether or not floral mite transfer occurs often in nature, mites infesting managed colonies may be experiencing selection for higher virulence due to high rates of horizontal mite transmission from crowding bees into apiaries (leading to high rates of drift and robbing) and from beekeeping practices that may facilitate mite transmission (such as the moving of brood from one colony to another). Research has shown that closely spaced colonies appear to share *Varroa* via drift or robbing from heavily infested colonies, but that such pronounced *Varroa* spread does not occur between colonies spaced more widely apart [36], as is typical in the wild. Thus, it is unclear whether mites in unmanaged (wild) colonies may evolve avirulence or virulence in the absence of human interference, and virulence theory can only offer accurate predictions if we first understand all mechanisms of mite transmission between colonies and their relative importance in the spread of mites through susceptible host populations.

Some risks demonstrated by these data have immediate relevance. For instance, the Isle of Man was declared a *Varroa*-free region by the European Union’s Department of the Environment, Food, and Agriculture in early 2015 [37], but by early 2016 reports of illegally imported and likely *Varroa*-contaminated bees called the safety of the Manx bees into question. Likewise, Newfoundland, Iceland, and a number of other island regions are currently considered free from *Varroa*. Great efforts have been taken to protect Australian beekeeping operations from invasion by *Varroa destructor*, with apparent success thus far [38]. Any region free from the parasite should carefully consider both the movement of bees and bee products, but also potentially flowers and other substrates on which *Varroa* could survive transport and which may subsequently attract honey bee foragers. Even regions already containing *Varroa* should consider any avenue of horizontal transmission that might allow locally absent bee viruses or acaricide resistance genes to be spread by a foreign mite to local bees. Though our data do not demonstrate that *Varroa* transmission on forage is a common occurrence, we have demonstrated the ease with which a floral mite may breach biocontainment and infest a new region. Since every phoretic mite is female and may be pregnant, and since *Varroa* are highly resistant to the costs of inbreeding because mating normally takes place between siblings, only a single biocontainment breakdown is required for *Varroa* to invade a new population of previously mite-free bees.

## Supporting Information

S1 Video A recording of a floral mite infestation. In this video, a mite on a daisy can be seen responding to the arrival of a foraging bee, approaching and then mounting the bee, and then crawling along the bee’s abdomen to the space between the bee’s abdomen and thorax.
(MP4)

S1 Behavior Dataset All behavioral observations analyzed in this manuscript are included as a digital spreadsheet document. (XLSX)
